# The impact of intracranial artery stenosis on the prognosis of non-dialysis patients with chronic kidney disease

**DOI:** 10.3389/fneur.2026.1772858

**Published:** 2026-03-18

**Authors:** Yinping Li, Zhenxing Fan, Na Lin, Yinghui Deng, Aihua Zhang

**Affiliations:** 1 Department of Nephrology, Xuanwu Hospital, Capital Medical University, Beijing, China; 2 National Clinical Research Center for Geriatric Diseases, Xuanwu Hospital, Capital Medical University, Beijing, China; 3 Department of Geriatrics, Xuanwu Hospital, Capital Medical University, Beijing, China

**Keywords:** anterior circulation stenosis, chronic kidney disease, intracranial artery stenosis, posterior circulation stenosis, prevalence, prognosis, transcranial color-coded Doppler

## Abstract

**Objective:**

To investigate the impact of intracranial artery stenosis (ICAS) on the prognosis of patients with non-dialysis chronic kidney disease (CKD).

**Methods:**

We retrospectively analyzed data from non-dialysis CKD patients who were hospitalized in the Department of Nephrology, Xuanwu Hospital, Capital Medical University, from January 2018 to December 2023, and had undergone transcranial color-coded Doppler (TCCD) examination. Follow-up was conducted until July 2025, with all-cause mortality defined as the primary endpoint.

**Results:**

A total of 790 non-dialysis CKD patients were included in the final analysis, of whom 361 (45.7%) had ICAS. Multivariate logistic regression analysis identified advanced age (odds ratio [OR] = 1.029), hypertension (OR = 3.758), diabetes mellitus (OR = 2.504), elevated fibrinogen (OR = 1.263), and lower hemoglobin (OR = 0.987,) as independent correlated factors of ICAS in non-dialysis CKD patients (all *p* < 0.05). Kaplan–Meier survival curves showed that patients with ICAS had a lower cumulative survival rate (Log-rank test, χ^2^ = 47.963, *p* < 0.001). Subgroup analysis revealed that patients with anterior circulation stenosis (ACS), posterior circulation stenosis (PCS), or concurrent anterior and posterior circulation stenosis all had lower survival rates compared to those without ICAS (Log-rank test, χ^2^ = 60.096, *p* < 0.001). Multivariate Cox proportional hazards regression analysis demonstrated that advanced age (hazard ratio [HR] = 1.070, 95% CI 1.044–1.096), CKD stages 4–5 (HR = 2.866, 95% CI 1.551–5.296), chronic heart disease (HR = 1.657, 95% CI 1.029–2.666), PCS (HR = 2.538, 95% CI 1.252–5.145), and lower corrected serum calcium (HR = 0.220, 95% CI 0.050–0.969) were independent predictors of all-cause mortality in non-dialysis CKD patients (all *p* < 0.05).

**Conclusion:**

The prevalence of ICAS in non-dialysis CKD patients was 45.7%. Advanced age, hypertension, diabetes mellitus, elevated fibrinogen, and lower hemoglobin were independent correlated factors for ICAS. Advanced age, CKD stages 4–5, chronic heart disease, PCS, and lower corrected serum calcium were independent predictors of all-cause mortality in this population.

## Introduction

Ischemic stroke is one of the leading causes of death in patients with chronic kidney disease (CKD). Intracranial artery stenosis (ICAS) is a major etiology of ischemic stroke (1). The prevalence of ICAS exhibits ethnic disparities, with the highest proportion of stroke attributed to ICAS in Asian populations (2). Patients with ICAS face an elevated risk of stroke and recurrence; notably, mild-to-moderate asymptomatic ICAS is an independent risk factor for future ischemic stroke in otherwise healthy individuals (3). A subgroup analysis of a US community-based cohort study found that estimated glomerular filtration rate (eGFR) less than 60 mL/min ∙ 1.73 m^2^ in the elderly population was an independent influencing factor for ICAS (4).

Chronic kidney disease patients are burdened with non-traditional cardiovascular risk factors, including uremic toxins, renal anemia, bone mineral metabolism disorders, and vascular calcification (5). The comorbidity of ICAS further exacerbates adverse prognosis and complicates clinical management in CKD patients. However, the independent predictors of ICAS in CKD patients remain inconclusive, and large-scale studies investigating the prognostic impact of ICAS on CKD patients are currently lacking. Thus, this study holds clinical significance, as it may provide evidence-based guidance for the prevention and management of cerebrovascular diseases in CKD patients.

## Materials and methods

### Study population and methods

**Figure 1:**
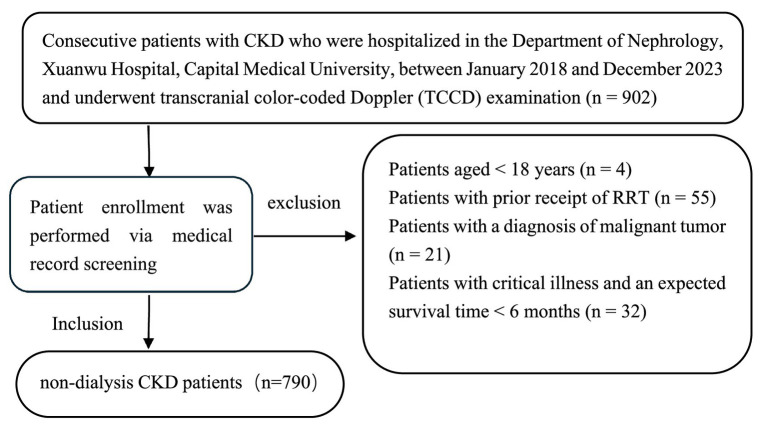
Study flowchart for participants screening.

Consecutive patients with CKD who were hospitalized in the Department of Nephrology of Xuanwu Hospital from January 2018 to December 2023 and all patients diagnosed with CKD underwent transcranial color-coded Doppler (TCCD) examination were enrolled. Exclusion criteria were as follows: (1) Age < 18 years; (2) Receipt of renal replacement therapy (RRT) at baseline; (3) Diagnosis of malignant tumor; (4) Critical illness with an expected survival time of < 6 months (see Figure 1).

A total of 790 participants met the predefined inclusion criteria. The follow-up cutoff date for all patients was July 2025. Sixty eight participants were lost to follow-up. This retrospective study was approved by the Institutional Review Board (IRB) of Xuanwu Hospital, Capital Medical University (Approval No. [2019]130). Patient data were de-identified to protect privacy, and an informed consent waiver was granted by the IRB due to the retrospective nature of the study.

Participants were stratified into two groups: non-intracranial artery stenosis (N-ICAS) and intracranial artery stenosis (ICAS). The ICAS group was further subcategorized based on: Stenosis severity: non-severe stenosis vs. severe stenosis; Stenosis location: anterior circulation stenosis, posterior circulation stenosis, and concurrent anterior–posterior circulation stenosis.

### Data collection

Demographic characteristics and comorbid medical history of included patients were extracted from electronic medical records. Baseline laboratory parameters (first measurements after admission) were collected, including: serum creatinine, serum albumin, serum calcium, serum phosphorus, serum potassium, total cholesterol (TC), low-density lipoprotein cholesterol (LDL-C), high-density lipoprotein cholesterol (HDL-C), intact parathyroid hormone (iPTH), fibrinogen, hemoglobin (Hb), white blood cell (WBC) count, lymphocyte count, and platelet count. Transcranial color-coded Doppler (TCCD) findings were reviewed by two independent vascular sonographers. The eGFR was calculated using the CKD-EPI equation, and corrected serum calcium was computed via the standard corrected calcium formula.

### Assessment of intracranial artery stenosis

1. Intracranial arteries evaluated by TCCD: anterior cerebral artery (ACA), middle cerebral artery (MCA), posterior cerebral artery (PCA), vertebral artery (VA), and basilar artery (BA).
2. TCCD-derived hemodynamic parameters: peak systolic velocity (PSV), end-diastolic velocity (EDV), mean flow velocity (MV), and vascular pulsatility index (PI).
3. Grading of intracranial artery stenosis via TCCD: Stenosis severity was classified as mild (30–49%), moderate (50–69%), severe (70–99%) and occlusion based on a composite analysis of hemodynamic indices (flow velocity, PI) and spectral morphology. In cases of multivessel stenosis, the severity was defined by the most severely stenosed artery.
4. Anatomical classification of intracranial stenosis:

Anterior circulation stenosis (ACS): stenosis involving the ACA and/or MCA;

Posterior circulation stenosis (PCS): stenosis involving the PCA, and/or VA, and/or BA.

### Definition of chronic heart disease

Organic heart diseases included coronary atherosclerotic heart disease (CAD), heart failure (HF), valvular heart disease (VHD), and cardiomyopathy; but arrhythmias (e.g., atrial fibrillation) were excluded.

### Definition of cerebrovascular disease

Ischemic cerebrovascular disease was defined as a history of stroke symptoms confirmed by head computed tomography (CT) or magnetic resonance imaging (MRI), with a definitive diagnosis made by a neurologist; but hemorrhagic cerebrovascular disease was excluded.

### Follow-up and study endpoint

Follow-up duration: All patients were followed up until July 2025, with a maximum follow-up time of 89 months and a mean follow-up time of 49.9 months.

Primary endpoint: All-cause mortality.

Follow-up modalities: Outpatient clinic visits, internet consultations, or telephone contacts.

Lost-to-follow-up (LTFU): Patients were classified as LTFU if they were lost to contact and no further clinical data could be obtained.

### Statistical analysis

Data analysis was performed using SPSS 26.0 software (IBM Corp.). Continuous variables: Normally distributed variables with homogeneous variance were expressed as mean ± standard deviation (±SD). Between-group comparisons were conducted using independent-samples *t*-test (two groups) or one-way analysis of variance (ANOVA) (three or more groups), with Least Significant Difference (LSD) *post-hoc* test for pairwise comparisons. Non-normally distributed variables were presented as median (interquartile range, IQR) [M (P25, P75)], and comparisons among multiple groups were performed using the Kruskal-Wallis H test; Categorical variables: Expressed as counts (percentages, %), with between-group comparisons using the Pearson’s chi-square test. For multiple pairwise comparisons, the Bonferroni correction was applied to adjust for type I error. Identified using multivariate logistic regression analysis after adjusting for potential confounders (variables with *p* < 0.1 in univariate analysis). Kaplan–Meier curves were generated to estimate survival probabilities, and differences in survival rates between groups were compared using the Log-rank test. Multivariate Cox proportional hazards regression model was used to assess independent risk factors for all-cause mortality. A two-tailed *p* value < 0.05 was considered statistically significant.

## Results

### Baseline characteristics of 790 non-dialysis CKD patients

The patients had a mean age of 60.30 ± 13.38 years, with a male-to-female ratio of 469/321. Among them, 597 cases (75.6%) were in CKD stages 1–3 and 193 cases (24.4%) in CKD stages 4–5. Comorbidities included hypertension (688 cases, 87.1%), diabetes mellitus (437 cases, 55.3%), cerebrovascular disease (178 cases, 22.5%), and chronic heart disease (207 cases, 26.2%). The etiologies of CKD were diabetic nephropathy (312 cases, 39.5%), hypertensive nephropathy (235 cases, 29.7%), chronic glomerulonephritis (191 cases, 24.2%), and others (52 cases, 6.6%) (Table 1)

**Table 1:** Comparison of baseline data between ICAS group and N-ICAS group.

Variables	The total study population (*n* = 790)	Non-intracranial artery stenosis group (N-ICAS) (*n* = 429, 54.3%)	Intracranial artery stenosis group (ICAS) (*n* = 361, 45.7%)	*t*/χ^2^/*Z*	*P*
Age (years)	60.30 ± 13.38	57.62 ± 13.91	63.49 ± 12.00	−6.295	<0.001
Male/female (*n*)	469/321	240/189	229/132	4.560	0.033
Serum creatinine (μmol/l)	156.17 (66.00, 185.25)	130.43 (60.00, 132.00)	186.75 (84.00, 231.50)	−7.626	<0.001
eGFR (ml/min∙1.73m^2^)	61.88 (30.62, 94.30)	71.60 (42.99, 99.39)	50.33 (22.41, 78.54)	−8.480	<0.001
CKD stage [*n* (%)]				39.492	<0.001
CKD stage 1–3 [*n* (%)]	597 (75.6)	362 (84.4)	235 (65.1)		
CKD stage 4–5 [*n* (%)]	193 (24.4)	67 (15.6)	126 (34.9)		
CKD Etiological classification [*n* (%)]				58.346	<0.001
Chronic glomerulonephritis	191 (24.2)	128 (29.8)	63 (17.5)		
Diabetic nephropathy	312 (39.5)	118 (27.5)	194 (53.7)		
Hypertensive renal damage	235 (29.7)	146 (34.0)	89 (24.7)		
Others	52 (6.6)	37 (8.6)	15 (4.2)		
Hypertension [*n* (%)]	688 (87.1)	356 (83.0)	332 (92.0)	14.069	<0.001
Diabetes mellitus [*n* (%)]	437 (55.3)	180 (42.0)	257 (71.1)	67.777	<0.001
Cerebrovascular disease [*n* (%)]	178 (22.5)	72 (16.8)	106 (29.4)	17.773	<0.001
Chronic heart disease [*n* (%)]	207 (26.2)	85 (19.8)	122 (33.8)	19.818	<0.001
Atrial fibrillation [*n* (%)]	27 (3.4)	10 (2.3)	17 (4.7)	3.359	0.067
Hyperlipidemia [*n* (%)]	448 (56.7)	251 (58.5)	197 (54.6)	1.238	0.266
Hyperuricemia [*n* (%)]	325 (41.1)	164 (38.2)	161 (44.6)	3.285	0.070
WBC count (×10^9^/L)	6.92 ± 2.27	6.86 ± 2.40	6.99 ± 2.11	−0.807	0.420
Hemoglobin (g/L)	121.03 ± 24.81	127.35 ± 23.06	113.54 ± 24.76	8.100	<0.001
Platelet count (×10^9^/L)	228.30 ± 75.20	231.10 ± 72.75	224.98 ± 77.97	1.139	0.255
Lymphocyte count (×10^9^/L)	1.85 ± 0.67	1.91 ± 0.65	1.77 ± 0.68	3.039	0.002
Total cholesterol (mmol/L)	4.75 ± 1.57	4.85 ± 1.64	4.64 ± 1.47	1.939	0.053
LDL cholesterol (mmol/L)	2.93 ± 1.33	2.98 ± 1.41	2.87 ± 1.23	1.155	0.249
HDL cholesterol (mmol/L)	1.17 ± 0.37	1.20 ± 0.39	1.12 ± 0.35	2.937	0.003
Fibrinogen (g/L)	4.28 ± 1.29	4.08 ± 1.24	4.53 ± 1.30	−4.860	<0.001
Serum albumin (g/L)	37.02 ± 8.32	38.00 ± 8.33	35.84 ± 8.16	3.669	<0.001
iPTH (ng/L)	87.37 (34.26, 102.05)	76.78 (31.73, 93.20)	97.52 (37.90, 116.80)	−3.414	0.001
Corrected serum calcium (mmol/L)	2.25 ± 0.17	2.26 ± 0.16	2.25 ± 0.18	0.589	0.556
Serum phosphorus (mmol/L)	1.25 ± 0.34	1.22 ± 0.33	1.29 ± 0.34	−2.937	0.003
Serum potassium (mmol/L)	4.15 ± 1.03	4.12 ± 1.30	4.19 ± 0.56	−1.015	0.311

### Comparison of baseline characteristics between ICAS group and N-ICAS group

The prevalence of ICAS was 45.7% (95% CI 42.1–48.9). Patients in the ICAS group were older, had a higher proportion of CKD stages 4–5, and a higher prevalence of comorbidities. Additionally, the ICAS group exhibited lower levels of hemoglobin, HDL, and serum albumin, as well as higher levels of fibrinogen, iPTH, and serum phosphorus, with all differences being statistically significant (*p* < 0.05) (Table 1).

### Comparison of baseline characteristics among patients with ACS, PCS, and concurrent anterior and posterior circulation stenosis

A total of 361 patients with ICAS were stratified into three subgroups according to the location of stenosis identified by transcranial color-coded Doppler (TCCD): the ACS group (*n* = 110, 13.9%), the PCS group (*n* = 93, 11.8%), and the concurrent anterior and posterior circulation stenosis group (*n* = 158, 20.0%). Patients in the PCS group exhibited the highest mean age. In contrast, the concurrent anterior and posterior circulation stenosis group was associated with the poorest renal function, the highest prevalence of hypertension, diabetes mellitus, and cerebrovascular disease comorbidities, and the highest proportion of severe stenosis (all *p* < 0.05) (Table 2)

**Table 2:** Baseline characteristics of patients with ACS, PCS, and concurrent anterior and posterior circulation stenosis (*n* = 361).

Variable	The ACS group (*n* = 110, 30.4%)	The PCS group (*n* = 93, 25.8%)	The concurrent anterior and posterior circulation stenosis group (*n* = 158, 43.8%)	*t*/χ^2^/*Z*/*H*	*P*
Age (years)	59.72 ± 12.46	67.15 ± 11.87^a^	63.97 ± 11.03^a^^b^	10.406	<0.001
Male/female (*n*)	75/35	48/45^a^	106/52^b^	7.582	0.023
Serum creatinine (μmol/l)	197.68 (82.50, 253.25)	147.47 (63.00, 159.00)	202.25 (98.25, 256.50)^b^	15.248	<0.001
eGFR (ml/min∙1.73m^2^)	53.11 (24.24, 82.50)	57.83 (29.65, 87.32)	43.97 (20.96, 64.37)^b^	11.540	0.003
CKD stage [*n* (%)]				9.298	0.010
CKD stage 1–3	75 (68.2)	70 (75.3)	90 (57.0)^b^		
CKD stage 4–5	35 (31.8)	23 (24.7)	68 (43.0)^b^		
CKD etiological classification [*n* (%)]				15.481	0.017
Chronic glomerulonephritis	25 (22.7)	16 (17.2)	22 (13.90)		
Diabetic nephropathy	54 (49.1)	40 (43.0)	100 (63.3)^b^		
Hypertensive renal damage	25 (22.7)	31 (33.3)	33 (20.9)		
Others	6 (5.5)	6 (6.5)	3 (1.9)		
Hypertension [*n* (%)]	102 (92.7)	80 (86.0)	150 (94.9)^b^	6.422	0.040
Diabetes mellitus [*n* (%)]	70 (63.6)	57 (61.3)	130 (82.3)^a^^b^	16.976	<0.001
Cerebrovascular disease [*n* (%)]	25 (22.7)	18 (19.4)	63 (39.9)^a^^b^	15.242	<0.001
ICAS severe stenosis [*n* (%)]	13 (11.8)	25 (26.9)^a^	75 (47.5)^a^^b^	39.467	<0.001
Chronic heart disease [*n* (%)]	30 (27.3)	35 (37.6)	57 (36.1)	3.072	0.215
Atrial fibrillation [*n* (%)]	4 (3.6)	3 (3.2)	10 (6.3)	1.662	0.436
Hyperlipidemia [*n* (%)]	59 (53.6)	56 (60.2)	82 (51.9)	1.689	0.430
Hemoglobin (g/L)	112.65 ± 23.54	122.12 ± 23.73^a^	109.11 ± 25.05^b^	8.519	<0.001
Platelet count (×10^9^/L)	235.57 ± 93.99	214.86 ± 61.51	223.56 ± 73.80	1.833	0.161
Lymphocyte count (×10^9^/L)	1.70 ± 0.62	1.87 ± 0.77	1.75 ± 0.66	1.759	0.174
LDL cholesterol (mmol/L)	2.91 ± 1.35	2.84 ± 0.91	2.86 ± 1.32	0.094	0.910
HDL cholesterol (mmol/L)	1.11 ± 0.32	1.22 ± 0.43^a^	1.08 ± 0. 31^b^	5.184	0.006
Fibrinogen (g/L)	4.74 ± 1.35	3.97 ± 1.08^a^	4.71 ± 1.31^b^	12.023	<0.001
Serum albumin (g/L)	35.12 ± 8.20	37.71 ± 7.45^a^	35.25 ± 8.41^b^	3.315	0.037
iPTH (ng/L)	93.69 (32.32, 114.45)	95.01 (37.18, 105.65)	101.43 (44.71, 130.55)	3.759	0.153
Corrected calcium (mmol/L)	2.25 ± 0.18	2.25 ± 0.17	2.25 ± 0.19	0.034	0.967
Serum phosphorus (mmol/L)	1.33 ± 0.32	1.22 ± 0.24^a^	1.31 ± 0.39^b^	3.298	0.038

a Indicates a statistically significant difference compared with the ACS group.

b Indicates a statistically significant difference compared with the PCS group.

All comparisons were adjusted using the Bonferroni correction. *P* < 0.05 was considered statistically significant.

### Analysis of independent correlated factors for ICAS in non-dialysis CKD patients

With reference to Table 1, variables were first subjected to univariate Logistic regression analysis. Variables with a univariate *p*-value < 0.1 were then included in the subsequent multivariate Logistic regression model. The results demonstrated that advanced age (OR = 1.029), comorbid hypertension (OR = 3.758), comorbid diabetes mellitus (OR = 2.504), reduced hemoglobin levels (OR = 0.987), and elevated fibrinogen levels (OR = 1.263) were independent predictors of ICAS in non-dialysis CKD patients (Table 3).

**Table 3:** Logistic regression analysis of predictors for ICAS in non-dialysis CKD patients (*n* = 790).

Variables	Univariate analysis		Multivariate analysis	
	OR (95% CI)	*P*	OR (95% CI)	*P*
Age (years)	1.035 (1.024 ~ 1.047)	<0.001	1.029 (1.010 ~ 1.048)	0.003
Gender (Male/Female)	1.366 (1.026 ~ 1.820)	0.033	1.494 (0.891 ~ 2.506)	0.128
CKD stages (4–5/1–3)	2.897 (2.064 ~ 4.065)	<0.001	1.399 (0.788 ~ 2.485)	0.251
hypertension (Yes/No)	2.348 (1.489 ~ 3.702)	<0.001	3.758 (1.505 ~ 9.382)	0.005
Diabetes mellitus (Yes/No)	3.418 (2.538 ~ 4.604)	<0.001	2.504 (1.596 ~ 3.927)	<0.001
Cerebrovascular disease (Yes/No)	2.061 (1.467 ~ 2.895)	<0.001	1.378 (0.839 ~ 2.263)	0.205
Chronic heart disease (Yes/No)	2.066 (1.497 ~ 2.852)	<0.001	1.189 (0.736 ~ 1.922)	0.478
Hyperlipidemia (Yes/No)	0.852 (0.642 ~ 1.130)	0.266		
Atrial fibrillation (Yes/No)	2.071 (0.936 ~ 4.581)	0.072	0.699 (0.203 ~ 2.401)	0.569
Hyperuricemia (Yes/No)	1.301 (0.979 ~ 1.729)	0.070	1.504 (0.968 ~ 2.337)	0.069
WBC count (×10^9^/L)	1.026 (0.964 ~ 1.091)	0.420		
Hemoglobin(g/l)	0.976 (0.970 ~ 0.982)	<0.001	0.987 (0.975 ~ 0.999)	0.029
Platelet count (×10^9^/L)	0.999 (0.997 ~ 1.001)	0.256		
Lymphocyte count (×10^9^/L)	0.717 (0.577 ~ 0.891)	0.003	0.809 (0.560 ~ 1.168)	0.259
Total cholesterol (mmol/L)	0.913 (0.833 ~ 1.002)	0.054	0.937 (0.799 ~ 1.100)	0.429
LDL cholesterol (mmol/L)	0.939 (0.843 ~ 1.045)	0.250		
HDL cholesterol (mmol/L)	0.558 (0.376 ~ 0.828)	0.004	0.551 (0.284 ~ 1.067)	0.077
Fibrinogen (g/L)	1.317 (1.175 ~ 1.478)	<0.001	1.263 (1.031 ~ 1.548)	0.024
Serum albumin (g/L)	0.969 (0.952 ~ 0.986)	<0.001	1.005 (0.974 ~ 1.037)	0.777
iPTH (ng/L)	1.003 (1.001 ~ 1.005)	0.012	1.000 (0.997 ~ 1.002)	0.766
Corrected serum calcium (mmol/L)	0.781 (0.344 ~ 1.774)	0.555		
Serum phosphorus (mmol/L)	1.963 (1.224 ~ 3.147)	0.005	0.998 (0.503 ~ 1.980)	0.996
Serum potassium (mmol/L)	1.077 (0.927 ~ 1.253)	0.332		

### Comparison of baseline characteristics between survivors and non-survivors

A total of 790 patients were followed up for a mean duration of 49.9 months, with 68 cases (8.6%) lost to follow-up and 111 cases (14.1%) deceased. Causes of death included cardiovascular disease (54 cases, 48.7%), cerebrovascular disease (21 cases, 18.9%), infectious diseases (14 cases, 12.6%), malignant tumors (8 cases, 7.2%), and other causes (14 cases, 12.6%). In the non-survivor group, 73.0% of patients had ICAS and 22.5% had severe ICAS, both of which were higher than those in the survivor group. Additionally, the non-survivor group exhibited older age, poorer renal function, higher proportions of comorbid diabetes, cerebrovascular disease, and chronic heart disease; elevated levels of fibrinogen, iPTH and serum phosphorus; and lower levels of hemoglobin, lymphocyte count, serum albumin, and corrected serum calcium compared with the survivor group (all *p* < 0.05) (Table 4)

**Table 4:** Comparison of baseline characteristics between the survival group and the non-survival group.

Variables	The total study population (*n* = 722)	Survival group (*n* = 611)	Non-survival group (*n* = 111)	*t*/χ^2^/*Z*	*P*
Age (years)	60.22 ± 13.52	58.51 ± 13.11	69.66 ± 11.75	−8.364	<0.001
Male/Female (*n*)	420/302	352/259	68/43	0.515	0.473
Serum creatinine (μmol/l)	151.16 (66.00, 178.25)	135.45 (63.00, 153.00)	237.69 (114.00,310.00)	−7.888	<0.001
eGFR (ml/min∙1.73m^2^)	62.79 (32.06, 94.48)	67.58 (39.52, 96.43)	36.38 (14.83, 51.38)	−8.810	<0.001
CKD stages [*n* (%)]				61.713	<0.001
CKD stages 1–3	554 (76.7)	501 (82.0)	53 (47.7)		
CKD stages 4–5	168 (23.3)	110 (18.0)	58 (52.3)		
CKD etiological classification [*n* (%)]				50.118	<0.001
Chronic glomerulonephritis	177 (24.5)	164 (26.8)	13 (11.7)		
Diabetic nephropathy	285 (39.5)	208 (34.0)	77 (69.4)		
Hypertensive renal damage	212 (29.4)	197 (32.2)	15 (13.5)		
Others	47 (6.5)	41 (6.7)	6 (5.4)		
Hypertension [*n* (%)]	629 (87.1)	527 (86.3)	102 (91.9)	2.663	0.103
Diabetes mellitus [*n* (%)]	393 (54.4)	310 (50.7)	83 (74.8)	21.884	<0.001
Cerebrovascular disease [*n* (%)]	154 (21.3)	118 (19.3)	36 (32.4)	9.636	0.002
Chronic heart disease [*n* (%)]	185 (25.6)	138 (22.6)	47 (42.3)	19.239	<0.001
Atrial fibrillation [*n* (%)]	25 (3.5)	19 (3.1)	6 (5.4)	1.481	0.224
Hyperlipidemia [*n* (%)]	415 (57.5)	358 (58.6)	57 (51.4)	2.015	0.156
ICAS [*n* (%)]	326 (45.2)	245 (40.1)	81 (73.0)	40.993	<0.001
Severe ICAS or occlusion [*n* (%)]	104 (14.4)	79 (12.9)	25 (22.5)	7.011	0.008
ICAS location				51.793	<0.001
ACS	105 (14.5)	83 (13.6)	22 (19.8)		
PCS	87 (12.0)	66 (10.8)	21 (18.9)		
Concurrent ACS and PCS	137 (19.0)	96 (15.7)	41 (36.9)		
Hemoglobin (g/L)	121.77 ± 25.03	125.10 ± 23.85	103.45 ± 23.48	8.817	<0.001
Platelet count (×10^9^/L)	228.42 ± 75.77	230.76 ± 74.72	215.59 ± 80.43	1.943	0.052
Lymphocyte count (×10^9^/L)	1.86 ± 0.68	1.90 ± 0.68	1.64 ± 0.66	3.753	<0.001
LDL cholesterol (mmol/L)	2.94 ± 1.32	2.94 ± 1.34	2.93 ± 1.24	0.038	0.969
HDL cholesterol (mmol/L)	1.17 ± 0.38	1.18 ± 0.38	1.14 ± 0.34	1.076	0.282
Fibrinogen (g/L)	4.25 ± 1.28	4.17 ± 1.27	4.68 ± 1.27	−3.858	<0.001
Serum albumin (g/L)	37.20 ± 8.27	37.62 ± 8.14	34.87 ± 8.59	3.244	0.001
iPTH (ng/L)	82.60 (33.74, 96.75)	75.62 (32.25, 88.35)	112.17 (54.98, 151.05)	−4.773	<0.001
Corrected serum calcium (mmol/L)	2.26 ± 0.17	2.27 ± 0.16	2.21 ± 0.23	2.344	0.021
Serum phosphorus (mmol/L)	1.25 ± 0.34	1.23 ± 0.34	1.34 ± 0.35	−2.950	0.003
Serum potassium (mmol/L)	4.15 ± 1.01	4.13 ± 1.07	4.29 ± 0.57	−1.604	0.109

### Impact of ICAS on prognosis in non-dialysis CKD patients

**Figure 2:**
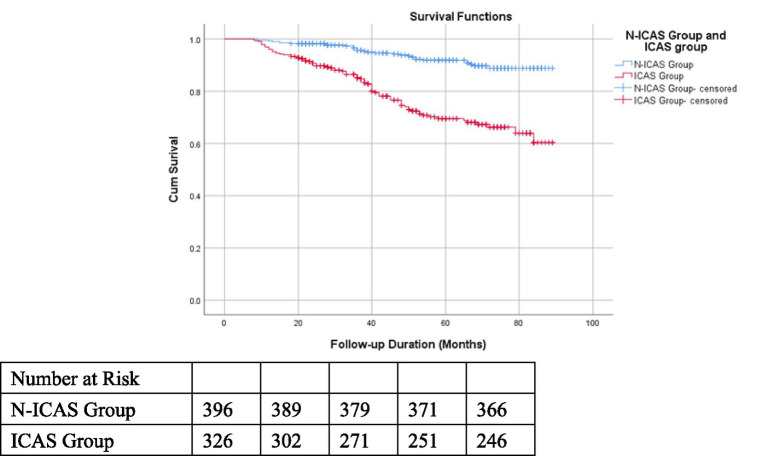
Kaplan–Meier survival curves of the ICAS group and N-ICAS group.

**Figure 3:**
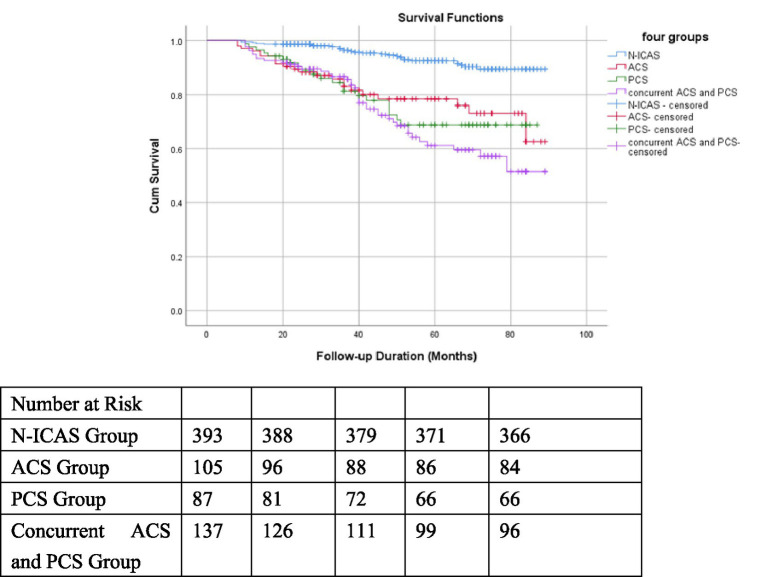
Kaplan–Meier survival curves of four groups classified by ACS and/or PCS.

Kaplan–Meier survival curves were constructed for the ICAS group and N-ICAS group: the cumulative survival rate was significantly lower in the ICAS group than in the N-ICAS group (Log-rank test, χ^2^ = 47.963, *p* < 0.001; Figure 2). Kaplan–Meier survival analysis stratified by the location of ICAS revealed that the cumulative survival rates in the ACS, PCS, and concurrent ACS and PCS subgroups were all significantly lower than those in the N-ICAS group (Log-rank test, χ^2^ = 60.096, *p* < 0.001; Figure 3). Additionally, Kaplan–Meier survival analysis based on the severity of ICAS showed no significant difference in cumulative survival rates among mild, moderate, and severe stenosis or occlusion subgroups (Log-rank test, χ^2^ = 0.370, *p* = 0.831).

### Independently predictors associated with all-cause mortality in non-dialysis CKD patients by Cox regressive analysis

We performed univariate Cox Analysis according to Table 4, then variables *p* < 0.1 in univariate Cox Analysis were selected into Multivariate Cox Analysis. Results of the multivariate Cox regression model showed that advanced age, CKD stages 4–5, comorbid chronic heart disease, PCS, and lower corrected serum calcium were independent predictors of all-cause mortality in non-dialysis CKD patients (*p* < 0.05) (Table 5).

**Table 5:** Cox regression analysis of factors associated with all-cause mortality in non-dialysis CKD patients (*n* = 722).

Variables	Univariate analysis		Multivariate analysis	
	HR (95% CI)	*P*	HR (95% CI)	*P*
Age (years)	1.074 (1.056 ~ 1.093)	<0.001	1.070 (1.044 ~ 1.096)	<0.001
Gender (Male/Female)	1.209 (0.825 ~ 1.772)	0.329		
CKD stage (4–5/1–3)	5.660 (3.882 ~ 8.252)	<0.001	2.866 (1.551 ~ 5.296)	0.001
Hypertension (Yes/No)	1.934 (0.978 ~ 3.824)	0.058	1.108 (0.369 ~ 3.323)	0.855
Diabetes mellitus (Yes/No)	3.162 (2.058 ~ 4.858)	<0.001	1.641 (0.938 ~ 2.871)	0.083
Cerebrovascular disease (Yes/No)	2.107 (1.414 ~ 3.140)	<0.001	1.515 (0.902 ~ 2.544)	0.116
Chronic heart disease (Yes/No)	2.370 (1.626 ~ 3.455)	<0.001	1.657 (1.029 ~ 2.666)	0.038
Hyperlipidemia (Yes/No)	0.883 (0.608 ~ 1.283)	0.514		
Atrial fibrillation (Yes/No)	1.514 (0.665 ~ 3.448)	0.323		
Hyperuricemia (Yes/No)	1.364 (0.937 ~ 1.986)	0.105		
Severe ICAS or occlusion (Yes/No)	1.904 (1.219 ~ 2.974)	0.005	0.517 (0.252 ~ 1.061)	0.072
WBC count (×10^9^/L)	0.959 (0.880 ~ 1.046)	0.349		
Hemoglobin (<110 g/L/≥110 g/l)	4.780 (3.257 ~ 7.017)	<0.001	1.199 (0.671 ~ 2.142)	0.539
Platelet count (×10^9^/L)	0.997 (0.994 ~ 1.000)	0.023	1.000 (0.997 ~ 1.003)	0.914
Lymphocyte count (×10^9^/L)	0.506 (0.365 ~ 0.700)	<0.001	0.665 (0.427 ~ 1.037)	0.072
Total cholesterol (mmol/L)	0.925 (0.808 ~ 1.058)	0.255		
LDL cholesterol (mmol/L)	1.000 (0.868 ~ 1.152)	0.999		
HDL cholesterol (mmol/L)	0.766 (0.450 ~ 1.302)	0.324		
Fibrinogen (g/L)	1.343 (1.189 ~ 1.516)	<0.001	1.175 (0.936 ~ 1.474)	0.164
Serum albumin (g/L)	0.956 (0.936 ~ 0.976)	<0.001	0.966 (0.931 ~ 1.002)	0.062
iPTH (ng/L)	1.002 (1.001 ~ 1.004)	0.002	1.000 (0.997 ~ 1.003)	0.942
Corrected serum calcium (mmol/L)	0.350 (0.125 ~ 0.979)	0.046	0.220 (0.050 ~ 0.969)	0.045
Serum phosphorus (mmol/L)	1.785 (1.319 ~ 2.416)	<0.001	1.033 (0.557 ~ 1.917)	0.918
Serum Potassium (mmol/L)	1.136 (1.025 ~ 1.260)	0.015	1.146 (0.877 ~ 1.499)	0.319
Four groups according to ACS and/or PCS				
N-ICAS	Reference group			
ACS	3.694 (2.103 ~ 6.489)	<0.001	2.014 (0.975 ~ 4.161)	0.059
PCS	4.166 (2.354 ~ 7.370)	<0.001	2.538 (1.252 ~ 5.145)	0.010
Concurrent ACS and PCS	5.394 (3.315 ~ 8.777)	<0.001	1.739 (0.833 ~ 3.628)	0.140

## Discussion

The prevalence of asymptomatic ICAS in the general population ranges from 6.0 to 24.5% (6, 7), whereas in patients with acute ischemic stroke, the prevalence of ICAS reaches 37.0 to 49.66%, accompanied by more severe symptoms and prolonged hospital stays (8, 9). Clinical research data on ICAS exhibit substantial variability, which can be attributed to differences in study populations, diagnostic modalities, and stenosis assessment criteria across studies (10). Digital subtraction angiography (DSA) serves as the gold standard for ICAS diagnosis; however, its invasive nature and the potential nephrotoxicity of contrast agents render it unsuitable for routine use in CKD patients. In contrast, transcranial color-coded Doppler (TCCD) is a non-invasive technique and is widely employed in clinical practice for evaluating ICAS in CKD patients.

In our study, we first demonstrated that the prevalence of ICAS in non-dialysis CKD patients was 45.7%, which was significantly higher than that in the general population. This discrepancy may stem from the fact that our study cohort consisted of hospitalized patients, who typically have more severe disease than outpatients, potentially leading to an overestimation of the overall ICAS prevalence in non-dialysis CKD patients. Asians are a population with a high prevalence of stroke (11), and CKD is recognized as a significant risk factor for stroke. Notably, patients with CKD have a higher mortality rate from stroke (12). Therefore, we consider that CKD patients presenting with proteinuria or renal insufficiency should undergo TCCD screening to evaluate the risk of ischemic cerebrovascular disease and mortality. All hospitalized patients in this study underwent TCCD examination rather than selectively enrolling a subset of CKD inpatients, which substantially mitigates potential selection bias.

In our study, 22.5% of patients had a history of ischemic stroke, with the proportion in the ICAS group being significantly higher than that in the N-ICAS group (29.4% vs. 16.8%). Patients in the ICAS group were older, had more comorbidities, and exhibited poorer renal function. A Chinese study enrolling 972 patients with acute ischemic stroke revealed that low eGFR was an independent predictor of new-onset ischemic stroke and transient ischemic attack (TIA) in patients with concurrent ICAS (13). Another study in China involving 3,954 individuals from the general population found that the prevalence of ICAS was 13.8%, with a much higher prevalence of ACS than PCS (11.9% vs. 4.2%) (14). In our study, the prevalence of ICAS was 45.7%, and the rates of ACS (13.9%), PCS (11.8%), and concurrent ACS and PCS (20.0%) were significantly higher than those in the general population. This finding may be attributed to the fact that CKD patients are at high risk of cardiovascular diseases (15), often presenting with cardiovascular risk factors such as hypertension and diabetes, and having poorer baseline health status.

Our study demonstrated that the ICAS group had lower HDL-C levels, while no differences in LDL-C and TC were observed between groups. Low HDL-C has been reported in the literature as an independent risk factor for cardiovascular diseases and is associated with stroke recurrence (16, 17). In the ICAS group, levels of iPTH and serum phosphorus were higher, while hemoglobin levels were lower. Both calcium-phosphorus metabolism disorders and renal anemia can exacerbate cardiovascular calcification (18). Vascular calcification is a risk factor for acute ischemic stroke (19, 20). However, in CKD patients lateral abdominal plain radiography is more commonly used to evaluate abdominal aortic calcification, whereas screening for cerebrovascular calcification is less frequent. Therefore, there is no information on cerebral artery calcification in our patients, which is worthy of further research in the future. Fibrinogen is a predictive marker for ischemic stroke, while elevated serum albumin levels are associated with a reduced risk of ischemic stroke (21). A Chinese cohort study involving 8,984 patients with acute stroke demonstrated that a higher fibrinogen-to-albumin ratio increased the risk of short-term and long-term adverse outcomes in patients with acute ischemic stroke (22). In our study, the ICAS group exhibited higher fibrinogen levels and lower serum albumin levels, suggesting an elevated risk of acute ischemic stroke.

Our Logistic multivariate regression analysis identified advanced age, comorbid hypertension, diabetes mellitus, lower hemoglobin levels, and higher fibrinogen levels as independent influencing factors for ICAS in non-dialysis CKD patients. Advanced age, hypertension, and diabetes mellitus are traditional risk factors, consistent with those for ICAS in patients with acute ischemic stroke (23, 24). Literature has reported that anemia significantly increases the risk of all-cause mortality and cardiovascular diseases in non-dialysis CKD patients (25, 26). In our study, hemoglobin was found to be a protective factor against ICAS in non-dialysis CKD patients, which may be attributed to chronic tissue hypoxia caused by anemia, leading to vascular endothelial dysfunction, promoting inflammatory oxidative stress, and inducing hemodynamic changes.

We enrolled non-dialysis CKD patients as the study population, and the prevalence of ICAS was 45.7%, significantly higher than that in the general population. However, we did not observe CKD stages 4–5 as an independent influencing factor for ICAS. The possible reasons may be as follows: CKD and ICAS share common risk factors, including hypertension, diabetes mellitus, and hyperlipidemia et al. These factors induce vascular endothelial injury and lipid deposition in the early stages of CKD, triggering ICAS formation. Since such injury persists throughout the progression of CKD, the differences between CKD stages are attenuated. Additionally, CKD is associated with risk factors such as uremic toxins, calcium-phosphorus metabolism disorders, microinflammatory status, metastatic calcification, and renal anemia, which exacerbate vascular endothelial injury and promote ICAS formation. Therefore, CKD staging alone cannot effectively predict the risk of ICAS in CKD patients.

Our follow-up revealed that the proportion of patients with ICAS in the deceased group was significantly higher than that in the survival group (73.0% vs. 40.1%), which was consistent with the findings in our Kaplan–Meier (K-M) survival curve analysis.

We observed that patients in the deceased group were older, had a higher prevalence of comorbidities including diabetes mellitus, chronic heart disease, and cerebrovascular disease, poorer renal function, and a higher proportion of diabetic nephropathy. Their hemoglobin and serum albumin levels were lower, suggesting a poorer nutritional status; their lymphocyte count was significantly lower than that of the survival group, indicating a poorer cellular immune function; their iPTH and serum phosphorus levels were higher, and their corrected serum calcium levels were lower, suggesting more calcium and phosphorus metabolism abnormalities.

The retrospective study on mechanical thrombectomy for patients with ischemic stroke revealed that CKD reduced the probability of favorable outcomes at 90 days post-stroke and was associated with an increased mortality rate (27). As ICAS is a high-risk factor for stroke, and cardiovascular disease is the leading causes of death in CKD patients, cerebrovascular assessment is crucial when evaluating the prognosis of CKD patients. This assessment should not only include the severity of ICAS but also the localization of ICAS lesions.

Multivariate Cox regression analysis identified PCS as an independent predictor of all-cause mortality in CKD patients. This may be attributed to the anatomical characteristics of intracranial arteries: the posterior circulation supplies vital centers such as the brainstem and thalamus, and its collateral circulation compensation capacity is inferior to that of the anterior circulation, making PCS more likely to induce fatal stroke. Additionally, the PCS group had a significantly higher proportion of severe ICAS than the ACS group. However, this is a retrospective study involving hospitalized patients, and it is not yet possible to determine a definite causal relationship between PSC and mortality in non-dialysis CKD patients. Further studies with larger sample sizes and prospective cohorts are needed to confirm this.

Based on the TCCD results, we classified ICAS into three groups: mild stenosis, moderate stenosis and severe stenosis or occlusion. The K-M survival curves for these three groups showed no significant differences in cumulative survival. Multivariate Cox regression also did not identify severe ICAS as an independent risk factor for all-cause mortality in non-dialysis CKD patients.

Multivariate Cox regression analysis also failed to identify severe ICAS as an independent risk factor for all-cause mortality in non-dialysis CKD patients. This observation may be attributed to the high burden of cardiovascular risk factors in CKD patients, where the prognostic weight of other comorbidities or risk factors outweighs the impact of ICAS severity. Further investigations with an expanded sample size and additional outcome parameters are warranted to clarify this relationship.

## Conclusion

In conclusion, this large-sample study is the first time to demonstrate that the prevalence of ICAS in non-dialysis CKD patients is 45.7%. The cumulative survival rate of the ICAS group was significantly lower than that of the N-ICAS group. Multivariate Cox regression analysis identified advanced age, CKD stages 4–5, chronic heart disease, posterior circulation stenosis, and lower corrected serum calcium as independent predictors of all-cause mortality in CKD patients.

## Data Availability

The original contributions presented in the study are included in the article/supplementary material, further inquiries can be directed to the corresponding author.
